# Stable trimer formation of spike protein from porcine epidemic diarrhea virus improves the efficiency of secretory production in silkworms and induces neutralizing antibodies in mice

**DOI:** 10.1186/s13567-021-00971-5

**Published:** 2021-07-07

**Authors:** Akitsu Masuda, Jae Man Lee, Takeshi Miyata, Takeru Ebihara, Kohei Kakino, Masato Hino, Ryosuke Fujita, Hiroaki Mon, Takahiro Kusakabe

**Affiliations:** 1 Laboratory of Insect Genome Science, Kyushu University Graduate School of Bioresource and Bioenvironmental Sciences, Motooka 744, Nishi-ku, Fukuoka, 819-0395 Japan; 2 Laboratory of Creative Science for Insect Industries, Kyushu University Graduate School of Bioresource and Bioenvironmental Sciences, Motooka 744, Nishi-ku, Fukuoka, 819-0395 Japan; 3 Department of Biochemistry and Biotechnology, Faculty of Agriculture, Kagoshima University, 1-21-24 Korimoto, Kagoshima, 890-0065 Japan; 4 Laboratory of Sanitary Entomology, Kyushu University Graduate School of Bioresource and Bioenvironmental Sciences, Motooka 744, Nishi-ku, Fukuoka, 819-0395 Japan

**Keywords:** Porcine epidemic diarrhea virus, spike protein, trimeric motif, silkworm-baculovirus expression vector system, subunit vaccine

## Abstract

Porcine epidemic diarrhea virus (PEDV) is a highly infectious pathogen of watery diarrhea that causes serious economic loss to the swine industry worldwide. Especially because of the high mortality rate in neonatal piglets, a vaccine with less production cost and high protective effect against PEDV is desired. The intrinsically assembled homotrimer of spike (S) protein on the PEDV viral membrane contributing to the host cell entry is a target of vaccine development. In this study, we designed trimerized PEDV S protein for efficient production in the silkworm-baculovirus expression vector system (silkworm-BEVS) and evaluated its immunogenicity in the mouse. The genetic fusion of the trimeric motif improved the expression of S protein in silkworm-BEVS. A small-scale screening of silkworm strains to further improve the S protein productivity finally achieved the yield of about 2 mg from the 10 mL larval serum. Mouse immunization study demonstrated that the trimerized S protein could elicit strong humoral immunity, including the S protein-specific IgG in the serum. These sera contained neutralizing antibodies that can protect Vero cells from PEDV infection. These results demonstrated that silkworm-BEVS provides a platform for the production of trimeric S proteins, which are promising subunit vaccines against coronaviruses such as PEDV.

**Supplementary Information:**

The online version contains supplementary material available at 10.1186/s13567-021-00971-5.

## Introduction

Since its first observation in Europe in the 1970s, porcine epidemic diarrhea virus (PEDV) has spread worldwide due to its highly contagious nature and has been causing significant economic losses [1, 2] by causing watery diarrhea, vomiting, dehydration, and a high mortality rate in neonatal pigs [3]. PEDV has a single positive-stranded RNA genome and belongs to the genus *Alphacoronavirus* in the family *Coronaviridae*. Coronaviruses have spike protein (S), membrane protein (M), envelope protein (E), and nucleocapsid protein (N) as four major structural proteins in the budded enveloped viruses [3]. Among these, the highly glycosylated S protein, consisting of S1 and S2 domains, forms a homotrimer and plays an important role in facilitating host cell attachment and entry [4]. Coronaviruses use their S1 domain to bind to various receptors, such as porcine aminopeptidase N: APN (Transmissible gastroenteritis virus), angiotensin-converting enzyme 2: ACE2 (Human coronavirus NL63), carcinoembryonic antigen-cell adhesion molecule: CEACAM1, or dipeptidyl peptidase: DDP4 (MERS-CoV), transferrin receptor 1 (PEDV), on host cells and their S2 domain to fuse with the cell membrane [5–9]. Therefore, there are many reports that the neutralizing epitopes are in the S protein [10–13]. Notably, it has been reported that several neutralizing epitopes are concentrated in a region called the CO-26 K equivalent epitope (COE), which contains amino acid residues 499–638 [10, 14] as a priority candidate region. In addition, the N-terminal domain of the S protein, which is unique to the genus Alphacoronavirus, has been reported to play an important role in PEDV infection in the intestine, and this second candidate region is thought to have the ability to bind to a type of sialic acids unique to the intestine [15]. Furthermore, as a third candidate region, several linear neutralizing epitopes have been reported in the N-terminal region of the S2 domain [16].

To develop a safe and effective vaccine, a rational strategy is to use recombinant proteins targeting the S protein of PEDV. An immunological study has shown that the administration of recombinant S1 protein produced by cultured porcine cells to pregnant sows can protect newborns from PEDV infection through passive immunity [17]. Similarly, enveloped virus-like particles with S, M, and E proteins produced in cultured insect cells were able to induce neutralizing antibodies equivalent to those of inactivated PEDV vaccines, although their production efficiency was lower [18]. It is generally reported that protein complexes such as VLPs, which have complex and regular structures, can induce very strong immunity. If the native structure of the coronavirus S protein, which forms a trimer, could be stabilized, it would have stronger immunogenicity than the monomeric S1 protein, and structural stabilization would enable mass production. In this study, we used a baculovirus expression system using individual silkworms, which is useful as a mass expression system for such a complex recombinant protein. In addition, by trimer formation, the recombinant S protein is expected to have a structure similar to that on the PEDV envelope, and a selective neutralizing antibody will be inducted.

For the above reasons, we attempted to construct a stable expression system for the PEDV S protein by introducing exogenous trimerization motifs. Most trimeric motifs are coiled-coils that bundle several α-helices and are also present in the S2 region of coronaviruses. However, the trimeric motifs formed by the ectodomains of S proteins detached from the membrane are unstable, and thus exogenous trimeric motifs have been employed in the expression of S proteins of various coronavirus genera [19–21]. The previous studies reported that the fusion of T4 fibritin of bacteriophage T4 and yeast transcription factor GCN4 to S proteins enhanced its trimerization and stabilization [19, 22].

In this study, to develop a subunit vaccine, we designed several constructs suitable for the expression of PEDV S protein using the silkworm-baculovirus expression vector system (silkworm-BEVS). The silkworm-BEVS utilizing recombinant *Bombyx mori* nucleopolyhedrovirus (BmNPV) to express a protein of interest (POI) is a useful platform for manufacturing biopharmaceuticals [23]. Under the control of powerful polyhedrin promoter, a variety of POIs expressed from recombinant BmNPVs accumulate in silkworm larvae and pupae in large quantities [24–27].

In the silkworm-BEVS, the introduction of the trimerization domain was very effective for stable mass secretion expression, and the vaccine candidate molecules produced by this system were able to induce strong humoral immunity and neutralizing antibodies against PEDV in mice.

## Materials and methods

### Silkworm cells and strain

The NIAS-Bm-Oyanagi2 cell (BmO2: kindly provided from Dr Imanishi) was maintained in IPL41 insect medium (Sigma, St. Louis, MO, USA) with 10% fetal bovine serum (Gibco, Grand Island, NY, USA) at 27 °C. The silkworm strains used in this study were provided by the Institute of Genetic Resources at Kyushu University, and the silkworm larvae were reared on mulberry leaves at 24–29 °C.

### Transmembrane prediction of PEDV S protein

The transmembrane domain of S protein from the PEDV 83P-5 strain (GenBank accession number, AB548621.1) was predicted using TMHMM v.2.0 software [28] or SOSUI software [29].

### Plasmid construction

The full-length spike gene from the PEDV 83P-5 strain was chemically synthesized [30]. The DNA fragment encoding amino acid 1–1332 of the S protein was amplified by PCR and subcloned into a modified pENTR11 vector containing a lobster L21 sequence (translation enhancer) at the N-terminal and affinity tags at the C-terminal in the original pENTR11 vector [24]. All primers and the PCR products for the constructs produced in this study are listed in Table 1. The resulting construct was named pENTR11L21-PEDV/S(1–1332) + TEVdH8S. pENTR11L21-PEDV/S(1–1332) + Tags(TEVdH8SKtag) in which Ktag (MKHKGS) is inserted into the pENTR11L21-PEDV/S(1–1332) + TEVdH8S was prepared by inverse PCR. Also, pENTR11L21-PEDV/S(1–1320) + Tags was prepared by inverse PCR with the pENTR11L21-PEDV/S(1–1332) + Tags as the template. In this study, trimerization motif from chicken cartilage matrix protein (CMP) was inserted into the C-terminal of the S ectodomain. The DNA fragment for the CMP trimerization motif was amplified by PCR using the chemically synthesized DNA coding the motif as a template [31]. The vector backbone was amplified from the pENTR11L21-PEDV/S(1–1332) + Tags by PCR and named PEDV/S(1–1320)-Tags-gib. These PCR products were assembled using the Gibson assembly method to generate pENTR11L21-PEDV/S(1–1320) + CMP + Tags vectors.

**Table 1:** **Primer sequences and the names of PCR products and generated vectors used in this study**

Primer name	Sequence (5ʹ to 3ʹ)	PCR product name	Vector name
S1-1332-noATG-F	ACGCCTTTAATTTACTTCTGGTTGTTCTTAC	PEDV/S(1–1332)-XhoI	pENTR11L21-PEDV/S(1–1332) + TEVdH8S
S1-1332-stopXhoI-F	GGGGCTCGAGATAAAAATAATCAACCAAACCCAC		
S1-1332-F	AAGCACAAAGGAAGCTAGACCCAGCTTTCTTGTACAAAG	PEDV/S(1–1332)-Tags	pENTR11L21-PEDV/S(1–1332) + Tags
S1-1332-R	CATGCTGCCCCCTCCATGATGATGGTGATGATGCTTTTCG		
S1-1320-F	TCGAGTGCAGGCGAAAACCTGTACTTCC	PEDV/S(1–1320)-Tags	pENTR11L21-PEDV/S(1–1320) + Tags
S1-1320-R	GATATATGTCTCAACTCGGTTGAGCCACTCAAGG		
PEDVS-TEV-F	TCGAGTGCAGGCGAAAACCTGTACTTCC	PEDV/S(1–1320)-Tags-gib	pENTR11L21-PEDV/S(1–1320) + CMP + Tags
S+CMP-R	GATATATGTCTCAACTCGGTTGAGCCAC		
CMP-F	CCGAGTTGAGACATATATCGAAGAAGACCCGTGTGAGTGTAAAAGC	CMP	
CMP-R	GGTTTTCGCCTGCACTCGAGATAATTTTATTCTCCAGCGCTTCAATGCG		

### Preparation of recombinant baculovirus

Each entry clone for PEDV S protein and pDEST8 vector (Invitrogen, Carlsbad, CA, USA) were subjected to gateway LR reaction according to the manufacturer’s protocol to generate the transfer plasmid for production of recombinant baculovirus. According to the protocols described previously, the transfer plasmid was introduced into *E. coli* DH10bac to generate recombinant BmNPV T3 bacmid by Tn7-mediated transposition [32]. In this study, the modified BmNPV T3 bacmid deleting six dispensable genes (chitinase A, cathepsin, egt, p26, p10, and p74) was used from our laboratory stock to improve protein expression [26, 33–35]. The recombinant baculoviruses were generated by introducing these bacmids into silkworm BmO2 cells and amplified by serial infection. The viral titers were determined by plaque assay.

### Expression analysis of PEDV spike protein in cultured insect cells and silkworm

Recombinant BmNPVs at an MOI of 1 were used for infection to 1 × 10^6^ BmO2 cells. The cell pellet and medium of infected cells were separated and collected at 4 days post-infection (dpi) by centrifugation at 1000 *g* for 10 min at 4 °C. After the centrifugation, the cell pellet was lysed with 1 mL of 1 × T buffer (20 mM Tris–HCl, 0.5 M NaCl, pH 7.5) and sonicated for 5 min by TOMY UD-100 (Tomy Seiko Tokyo, Japan) at 4 °C. After centrifugation at 9000 *g* for 10 min at 4 °C, the supernatant was collected, and the precipitated was resuspended with the same volume of 1 × Phosphate-buffered saline (PBS; 137 mM NaCl, 2.7 mM KCl, 10 mM Na_2_HPO_4_·12H_2_O, 1.8 mM KH_2_PO_4_, pH 7.4).

Silkworm larvae were injected with recombinant BmNPVs of 1 × 10^4^ plaque-forming unit per larva. The sera of infected larvae at 4 dpi were collected and centrifuged at 3200 *g* for 10 min at 4 °C. The supernatants were collected and stored at −80 °C until use.

### Purification of the PEDV spike protein

As for the large-scale purification, 10 mL of the larval serum from the silkworm infected with BmNPV-PEDV/S(1–1320) + CMP + Tags were thawed and mixed with 40 mL of buffer A (20 mM Tris–HCl, 0.5 M NaCl, pH 7.5). After sonication by TOMY UD-100 (Tomy Seiko) at 4 °C for 10 min, the sample was centrifuged at 12 110 *g* for 30 min at 4 °C. The supernatant was filtered using a 0.45 µm filter (Millipore, Milford, USA).

Two-step affinity chromatography was performed to purify the S protein. Briefly, the filtered sample was loaded onto a 5 mL HisTrap excel column (Cytiva, Tokyo, Japan) equilibrated with buffer A containing 20 mM imidazole followed by the column wash with buffer A containing 30 mM imidazole (25 mL). The bound proteins were eluted with buffer A containing 500 mM imidazole. The 25 mL of the eluent were pooled in a 50 mL tube. The elution containing PEDV S protein was ultrafiltrated using Amicon ultra-15 100 K filters (Merck, USA) to substitute the buffer to 1 × PBS. A final 50 mL diluted solution was applied to 5 mL STREP-tactin columns (IBA GmbH, Germany), followed by washing with 1 × PBS (25 mL) and elution with the buffer containing 2.5 mM desthiobiotin (25 mL). The eluent was concentrated by ultrafiltration using Amicon ultra-15 100 K filters (Merck) and dialyzed against 1 × PBS. The purified PEDV S protein was quantified by bradford assay using ImageJ software version 1.51 s using BSA as a standard.

### SDS-PAGE and Western blotting

The fivefold dilution of the larval serum and all other specimens were mixed with an equal volume of 2 × SDS sample buffer (0.1 M Tris–HCl pH 6.8, 0.2 M dithiothreitol, 4% SDS, 20% glycerol, and 0.02% bromophenol blue) and denatured at 96 °C for 10 min. Subsequently, samples were separated on 12% SDS-PAGE gels. After separation, gels were stained by Coomassie Brilliant Blue (CBB). In the Western blotting analysis, proteins separated by SDS-PAGE were transferred to PVDF membrane (Millipore). Membranes were treated with blocking buffer (Tris-buffered saline containing 5% skim milk) and incubated with HisProbe-HRP (Thermo Scientific, Waltham, USA) for 1 h at room temperature. The specific protein bands were visualized using the Super Signal West Pico Chemiluminescent Substrate (Thermo Scientific). In the comparison of the S protein expression among silkworm strains, the intensities of each specific band were measured using ImageJ software version 1.51 s, and the relative intensities were calculated by setting the mean intensity of f38 strain to 1.

### Blue-native PAGE

Protein samples were mixed with a 4 × NativePAGE sample buffer (Thermo Fisher Scientific, Waltham, MA, USA) and were electrophoresed in 3 to 12% Bis–Tris NativePAGE gel (Thermo Fisher Scientific) for 115 min at 150 V. As a cathode buffer, NativePAGE running buffer (Thermo Fisher Scientific) containing NativePAGE cathode buffer additive (Thermo Fisher Scientific) was used. NativePAGE running buffer was used as an anode buffer. NativeMark unstained protein standard (Thermo Fisher Scientific) was used as a size standard. The gels after electrophoresis were stained using CBB.

### Size-exclusion chromatography

Purified S protein was subjected to size-exclusion chromatography using a superose 6 increase 10/300 GL column (Cytiva) in a 1 × PBS buffer. Thyroglobulin (669 kDa), Ferritin (440 kDa), and Aldolase (158 kDa) were used as molecular weight markers.

### Dynamic light scattering (DLS)

The size distribution of PEDV S proteins was analyzed by dynamic light scattering using an ELSZ-2000ZS (Otsuka electronics CO., Ltd, Osaka, Japan). To assess the thermal stability, 0.6 mg/mL of the S protein were incubated at 4, 30, 40, 50, 60, 70, 80, 90 °C for 5 min and measured the size changes. The measurement was performed at room temperature. Before the heating, the sample was centrifuged at 15 000 rpm for 5 min to remove aggregates which interfere with obtaining precise spectra in DLS.

### Mouse immunization

A total of 6 female Balb/c mice (7-week-old) were inoculated with 30 μg of PEDV S protein with micro-emulsion adjuvant (Montanide IMS 1313, Seppic, France) and boosted at 14 and 28 days after prime vaccination. At 0, 21, and 42 days after prime vaccination, the sera were collected and used for antigen-specific IgG ELISA and neutralization assay.

### S protein-specific IgG response evaluation with Enzyme-linked immunosorbent assay

The 96-well ELISA plates (Sumilon type S, Sumitomobakelight co., ltd, Japan) were coated with 50 µL of 5 µg/mL of the S proteins in bicarbonate buffer (pH9.6) and incubated overnight at 4 °C. The plates were washed with PBS containing 0.1% Tween-20 (PBST) and PBS and blocked with a blocking buffer (PBS containing 1% BSA) at room temperature for 1 h. After washing, 50 µL of each serum sample was added into each well. The plates were washed, and 50 µL of 0.5% BSA containing a peroxidase-conjugated goat anti-mouse IgG Fab antibody (Jackson immunoresearch, Laboratories, Inc., West Grove, PA, USA) was added into each well and incubated for 1 h. Finally, the plates were washed with PBST and PBS, and the color reaction was developed with 1-Step Turbo TMB-ELISA substrate solution (Thermo Scientific). The optical density at 450 nm (OD450nm) was read using a microplate reader.

### Serum virus neutralization assay

Eagle MEM containing 10% fetal bovine serum (biosera, USA) and 50 µg/mL Gentamicin (Merck) was prepared as a growth medium for the neutralization assay. The Immune sera of each mouse were heated at 56 °C for 30 min. Then, 25 μL of serially diluted sera were mixed with an equal volume of 4000 TCID_50_/mL PEDV (P-5 V strain) and incubated in the 96 well plates at 37 °C for 1 h [36]. The plate was added 50 μL of Vero cells at 1 × 10^5^ cells/mL, and the cells were incubated in the growth medium at 37 °C with 5% CO_2_ for 5 days. The presence of the cytopathic effect (CPE) was checked, and the highest dilution factor at which CPE was not observed was used as the neutralizing antibody titer.

## Results

### Expression of PEDV S ectodomain in silkworm-BEVS

**Figure 1:**
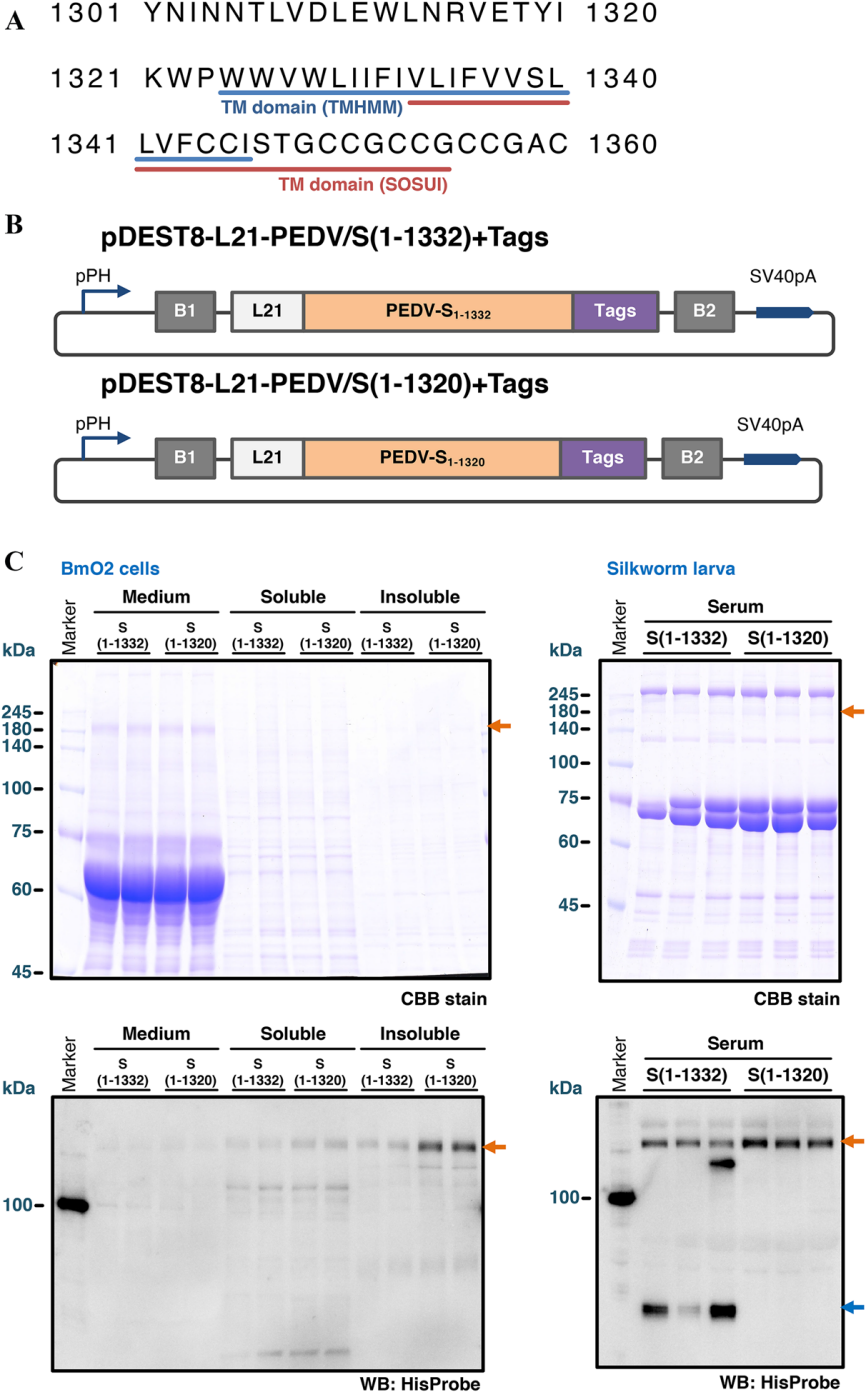
**Expression of the ectodomain of PEDV S protein in cultured silkworm cells and silkworm larva.** **A** Transmembrane (TM) domain prediction of PEDV S protein. The TM domains predicted using TMHMM v.2.0 software and SOSUI software are indicated by a blue and red underline, respectively. **B** The constructions of baculovirus transfer vector. pPH: polyhedrin promoter; B1 and B2: recombination sites for Gateway cloning; L21: translational enhancer; Tags: TEV protease cleavage site, 8 × histidine tag, STREP(II) tag, 6 × histidine tag, and Ktag (MKHKGS); SV40pA: polyadenylation signal (SV40). **C** Expression of PEDV S(1–1320) and S(1–1332) protein in BmO2 cells (left panel) and silkworm larva (right panel). Medium: culture medium; Soluble: soluble samples of cell lysates; Insoluble: insoluble samples of cell lysates. All samples from BmO2 cells (5 μL) and silkworm sera (0.5 μL) were resolved on an 8% SDS-PAGE. The recombinant PEDV S protein was also detected by Western blotting using HisProbe-HRP. Orange and blue arrows indicate the PEDV S protein and the cleaved products, respectively.

In order to secrete PEDV S protein into silkworm serum, the transmembrane domain (TM domain) predicted were removed from the expression constructs. Two software programs, SOSUI and TMHMM v.2.0, predicted different amino acid regions 1333–1355 (Figure 1A, red line) and 1324–1346 (Figure 1A, blue line) as a TM domain of the S protein, respectively. Therefore, two constructs were designed based on the prediction and generated the recombinant BmNPVs expressing the candidates for S ectodomain, PEDV/S(1–1332) and PEDV/S(1–1320) as described under the Materials and Methods (Figure 1B). PEDV/S(1–1332) and PEDV/S(1–1320) correspond to S proteins without TM domains predicted by SOSUI and TMHMM v.2.0, respectively. The expression analysis of the ectodomain in BmO2 cells and silkworm larvae revealed that both constructs were secreted inefficiently into the culture media and larval sera, respectively (Figure 1C). The TM domain is well conserved among coronaviruses, and in PEDV, residue 1321 corresponds to the beginning of the TM domain of other coronaviruses [37]. Therefore, it is likely that the S1-1320 construct, in which residues 1321 and later were removed, was more efficiently secreted. Importantly, a part of PEDV/S(1–1332) in the serum was cleaved probably by trypsin (Figure 1C). This construct partially retains the TM domain, and its presence on the membrane may make it more susceptible to cleavage by trypsin. In conclusion, 1–1320 residue of PEDV S protein was stably secreted into the silkworm serum without cleavage, but the expression levels were insufficient for developing an inexpensive vaccine.

### Construction and expression of PEDV S protein fused with CMP trimerization motif

**Figure 2:**
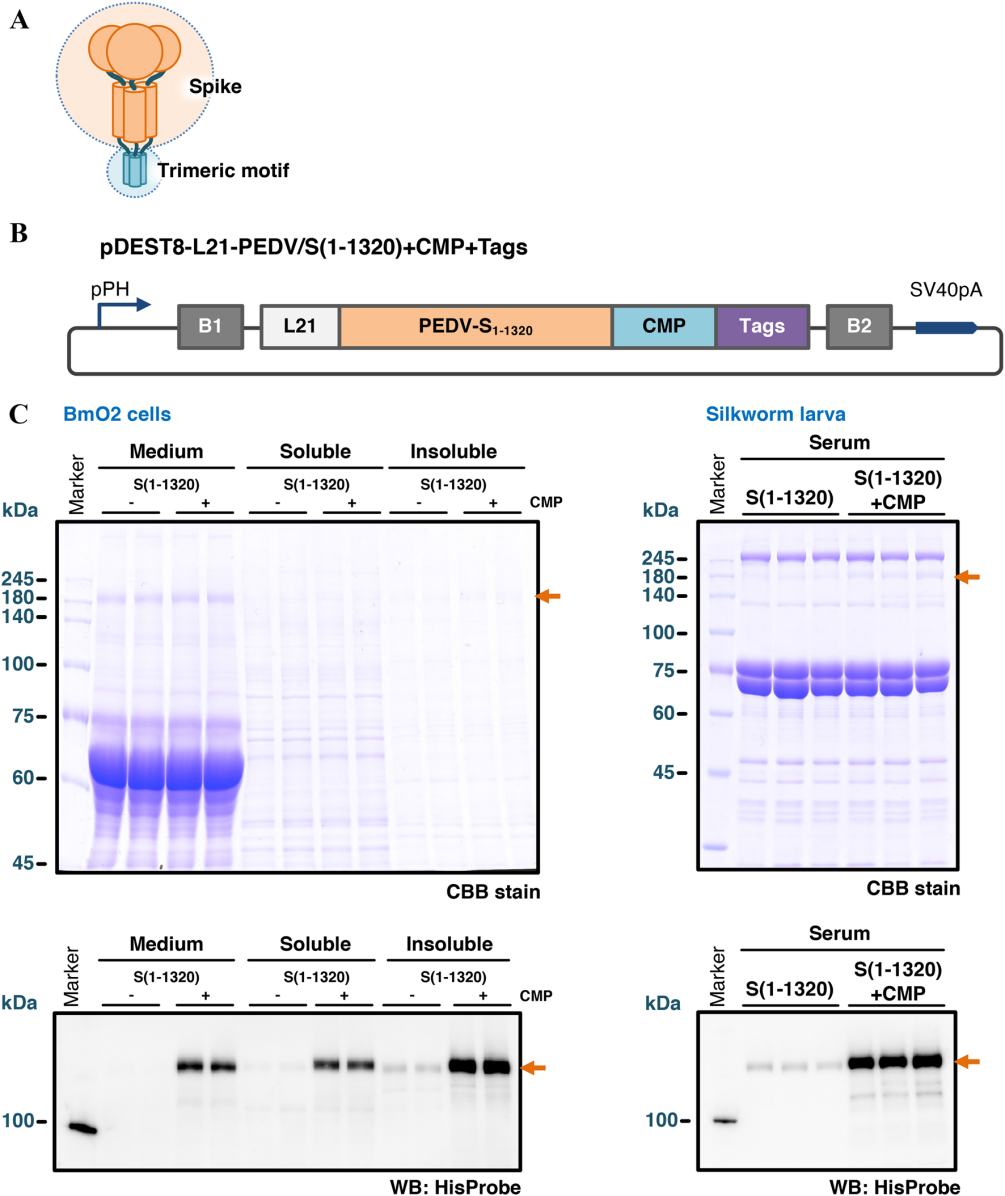
**Genetic fusion of trimerization motifs to the S protein.** **A**, **B** Scheme of PEDV S protein fused with the trimeric motif. The CMP coiled-coil was appended at the C terminus of the S protein. **C** Expression analysis of the S protein fused with the trimeric motif. Medium: culture medium; Soluble: soluble samples of cell lysates; Insoluble: insoluble samples of cell lysates. All samples from BmO2 cells (5 μL) and silkworm sera (0.5 μL) were resolved on an 8% SDS-PAGE. The recombinant PEDV S protein was also detected by Western blotting using HisProbe-HRP. Orange arrows indicate the PEDV S protein.

From the above results, it is assumed that PEDV S proteins are difficult to form homotrimer spontaneously in the insect cells. To improve secretion by forming stable homotrimer of PEDV S protein, we fused chicken cartilage matrix protein (CMP) to the S protein, following a previous study in which CMP was used to stabilize the trimeric antigen [38]. As described under the Materials and Methods, PEDV/S(1–1320) was C-terminally fused with the trimeric motif of CMP and generated the recombinant BmNPV (Figures 2A and B). The amino acid sequence of PEDV/S(1–1320) + CMP + Tags was shown in the Additional file 1. The expression analysis by Western blotting demonstrated that C-terminal fusion of CMP to the PEDV S ectodomain improved the secretion efficiency drastically compared to that without CMP (Figure 2C). In the CBB staining of polyacrylamide gel, the clear protein bands corresponded to PEDV S protein fused with CMP from 0.5 µL of the silkworm sera was detected, although at the same position, a non-specific band is also observed in the serum of silkworm expressing PEDV/S(1–1320) without CMP (Figure 2C).

### The expression levels of the PEDV S protein differed among silkworm strains

**Figure 3:**
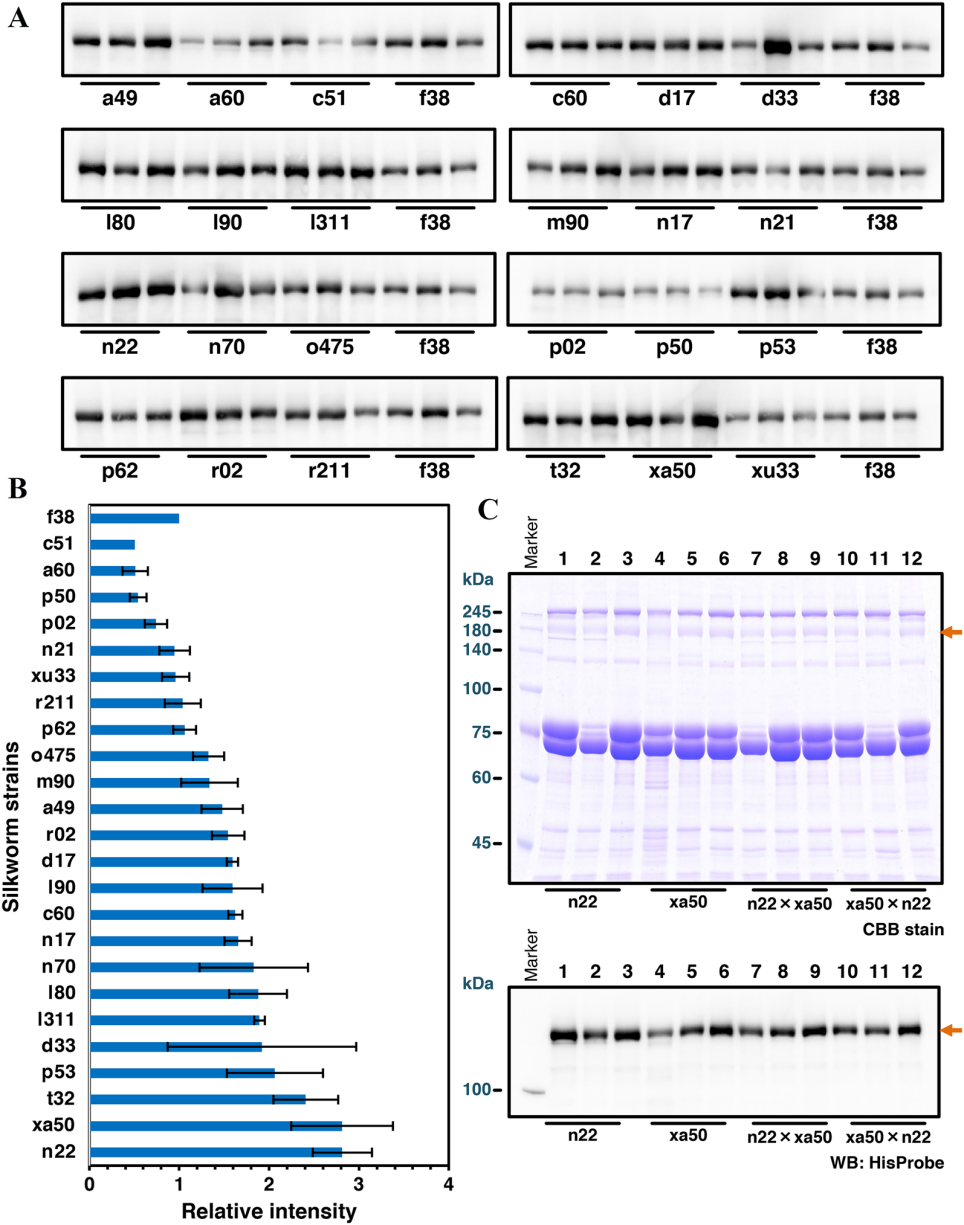
**Screening of the suitable silkworm strain for PEDV S protein expression.** **A** The Western blotting analysis of 25 silkworm strains infected with recombinant baculovirus encoding PEDV/S(1–1320) + CMP + Tags. The sera of 0.5 μL were subjected to 8% SDS-PAGE, and the specific band was detected using HisProbe-HRP. Three individual samples were prepared from each strain, and f38 sera were used as a control. **B** Comparison of relative band intensities among silkworm strains by Western blotting detecting PEDV/S(1–1320) + CMP + Tags protein. The intensities of each band were measured, and the relative intensities were calculated using the average band intensity of f38 strain as 1. Bars represent mean relative intensities ± SE (*N* = 3). **C** Expression analysis of the hybrid silkworm of n22 strain and xa50 strain. Three individual serum samples of each n22 (lane1-3), xa50 (lane4-6), and n22 × xa50 (lane7-9), and xa50 × n22 (lane10-12) were resolved in an 8% SDS-PAGE with CBB stain (upper panel) and Western blotting using HisProbe-HRP (lower panel). Orange arrows indicate the PEDV S protein.

To further improve the production of PEDV S protein, small-scale screening of the S protein expression using 25 silkworm strains was performed. The recombinant BmNPV expressing PEDV/S(1–1320) + CMP + Tags was injected into each larva, and the sera of three individuals infected were collected to analyze the S protein expression levels. Comparison of the specific bands by Western blotting analysis using HisProbe-HRP revealed that n22 and xa50 strains showed relatively high S protein expression (Figures 3A and B). The n22 and xa50 strains preserved in Kyushu University are inbred lines, and they are not suitable for mass-rearing, and the size of the last instar larvae is relatively small. Since these shortcomings can be overcome by utilizing hybrid vigor through F1 hybrid formation, we crossed n22 and xa50 strains (n22 × xa50 and xa50 × n22) and confirmed that the expression level of S protein was not altered. As shown in Figure 3C, the expression of S protein in serum of these hybrids infected with the recombinant BmNPV was comparable to those of n22 and xa50 strains. Although there was no synergistic effect in the amount of expression, the crossbreeding of these strains was easier to raise, and the volume of serum recovered from each larva was greatly increased.

### Characterization of the multimer formation of PEDV S protein fused with CMP

**Figure 4:**
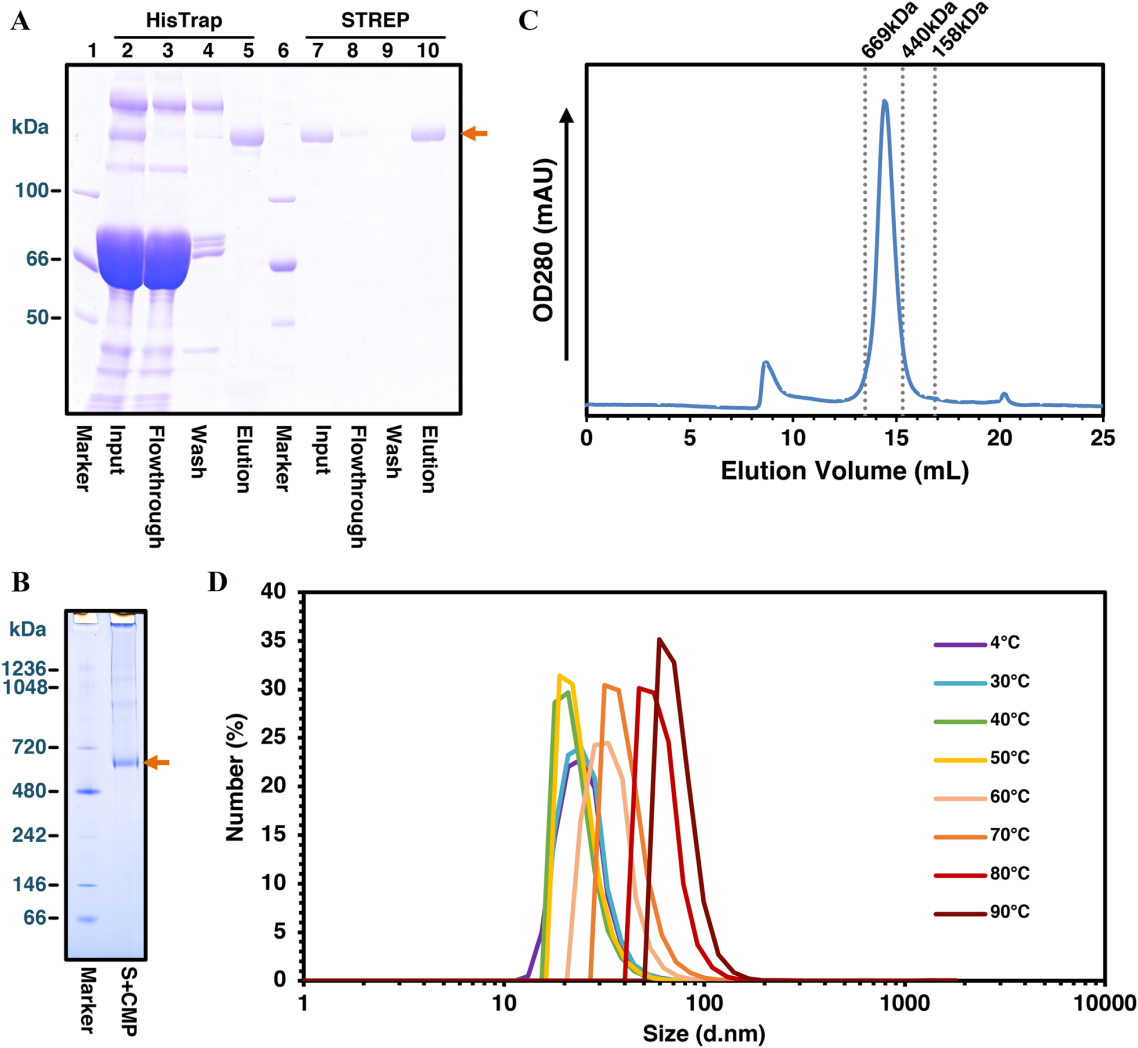
**Characterization of PEDV/S(1–1320) + CMP + Tags protein.** **A** Purification of the S protein from the silkworm serum. Two-step affinity chromatography using HisTrap excel column and STREP-tactin column was executed described in the Materials and Methods. Lanes 1 and 6, molecular weight marker; lanes 2–5, nickel affinity chromatography; lanes 7–10, STREP-tactin affinity chromatography. All samples were resolved in 8% SDS-PAGE and visualized by CBB staining. An orange arrow indicates the PEDV S protein. **B** Blue-Native PAGE analysis of the S protein. After electrophoresis, the gel was further stained by CBB. An orange arrow indicates the PEDV S protein trimer. **C** Size-exclusion chromatography of PEDV S protein. The chromatogram of the S protein is shown as a blue curve. The molecular weight markers are indicated as dashed lines. **D** The size changes of the S protein against heating. The thermal stability profile was collected by DLS.

The PEDV/S(1–1320) + CMP + Tags proteins were purified from the serum of silkworm (xa50 × n22) using two-step affinity chromatography as described under the Materials and Methods. As shown in Figure 4A, the S protein was appeared as a single band after the STREP purification, and the final yield from 10 mL sera was about 2 mg. To determine whether the S proteins form homotrimer, Blue-Native PAGE (BN-PAGE) and size-exclusion chromatography analyses were carried out. In the BN-PAGE analysis, a band was observed between 480 and 720 kDa of the molecular weight marker (Figure 4B). The molecular weight of PEDV/S(1–1320) + CMP + Tags protein monomer is calculated as 153 kDa, and that of the trimer is 459 kDa. Because of the post-translational modifications such as glycosylation, the molecular weight of PEDV/S(1–1320) + CMP + Tags protein monomer reached about 180 kDa from our SDS-PAGE analysis, and based on this calculation that of the trimer is expected to be about 540 kDa (Figure 2C). Consistent with this result, the peak of PEDV/S(1–1320) + CMP + Tags protein analyzed by size-exclusion chromatography was eluted between 440 and 669 kDa (Figure 4C), indicating that the PEDV S protein fused with CMP formed homotrimer. Finally, the thermal stability of PEDV/S(1–1320) + CMP + Tags homotrimer was analyzed by dynamic light scattering (DLS). PEDV/S(1–1320) + CMP + Tags had a long diameter of about 24 nm measured at 4 °C that is slightly longer than that observed in the cryo-EM [22]. The S protein showed the same diameter at 4, 30, 40, and 50 °C, but began to change from about 24 nm to 33 nm at 60 °C, and became even larger at 70 °C to 90 °C (Figure 4D). These results indicate that the PEDV/S(1–1320) + CMP + Tags protein purified from silkworm serum forms a stable trimer up to about 50 °C.

### PEDV S protein elicited humoral immunity in mice

**Figure 5:**
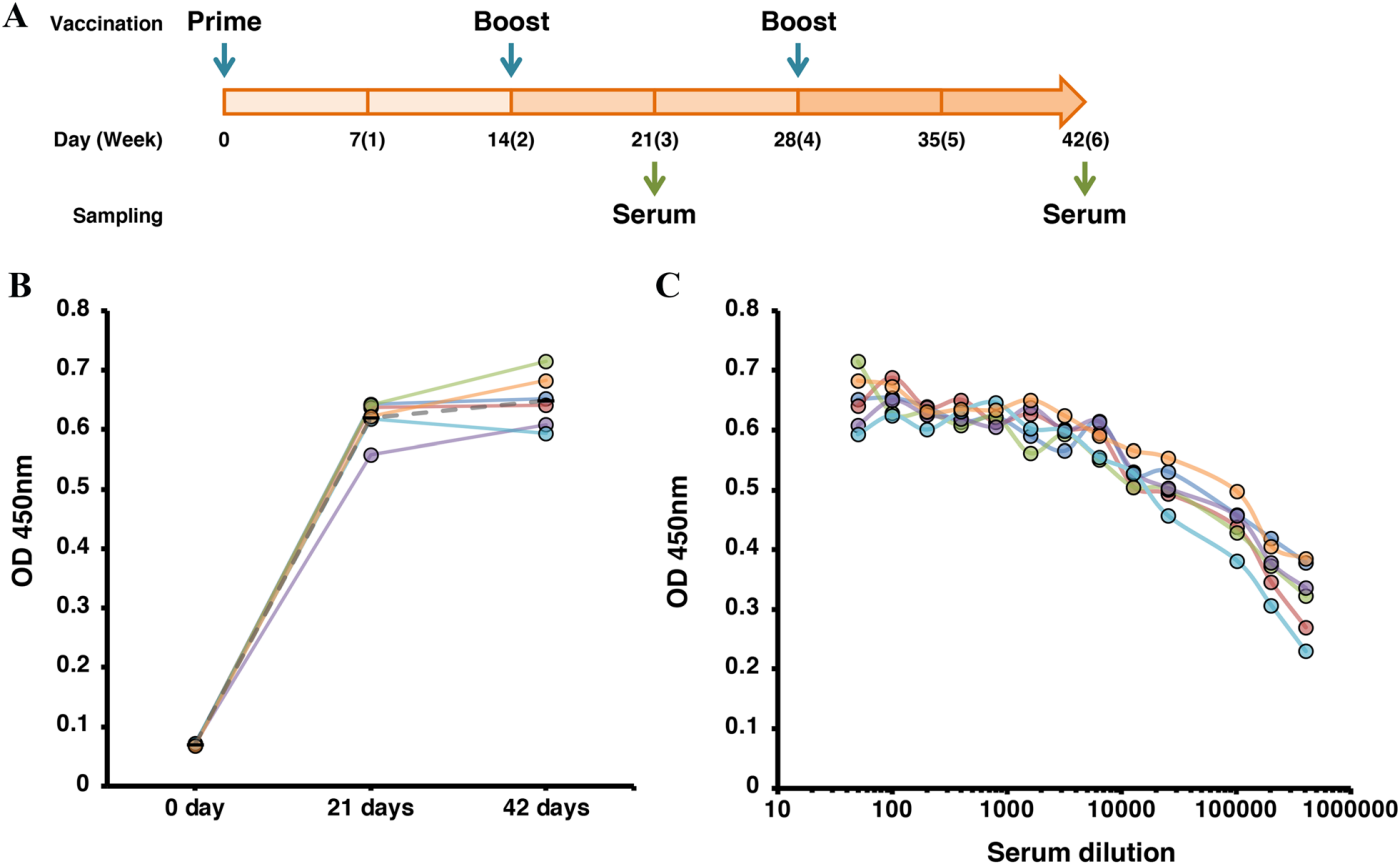
**Immunogenic study of the S protein in mice.** **A** Schematic representation of vaccination schedule in mice. Female mice (*N* = 6) were vaccinated with 30 μg of purified PEDV/S(1–1320) + CMP + Tags in a micro-emulsion adjuvant (IMS1313). The mice were boosted at 14- and 28-days post-vaccination (dpv), and serum samples were collected at 0, 21, and 42 dpv. **B** ELISA analysis to detect the increase of PEDV S protein-specific antibodies. All serum samples collected at each time point from each mouse were diluted at 1:50. The OD 450 nm values from each mouse serum are indicated as circles, and the average values are indicated as a bar. **C** ELISA analysis of the serum dilutions at 42 dpv. All serum samples are diluted from 1:50 to 1:404 800. The OD 450 nm values from each mouse are indicated.

Purified PEDV/S(1–1320) + CMP + Tags protein mixed with micro-emulsion adjuvant (Seppic, France) was inoculated three times into 6 female Balb/c mice at a dose of 30 µg, 14 days apart (Figure 5A). This administration condition was previously used to induce efficient antibody production in mice with a silkworm-derived vaccine antigen [26]. We decided to use this animal model because it is optimal to verify the induction of neutralizing antibodies. Serum samples at days 0, 21, and 42 after prime vaccination were collected and subjected to ELISA analysis to determine the induction of PEDV S protein-specific IgG antibodies. The ELISA analysis showed that the first boost vaccination, given 14 days after the prime vaccination, was sufficient to saturate the antibody production level, and the second boost vaccination did not enhance the antibody response (Figure 5B). The ELISA OD values of the immunized mice sera did not drop even at a high dilution factor (Figure 5C), and as shown in Additional file 2, the OD value did not drop as much as that of the negative control group, indicating that the S protein-specific antibody titer was higher than the maximum dilution factor in the experiment. High S protein-specific IgG antibody levels were induced even after two doses of 1 μg or 10 μg S protein moiety, suggesting that a sufficient immune response can be obtained even if the dose and number of doses are reduced (Additional file 2). A neutralization assay revealed that the S protein induced neutralizing antibodies against PEDV in the mice antiserum (Table 2). ﻿Collectively, we confirmed PEDV/S(1–1320) + CMP + Tags induced sufficient antibodies that can block PEDV infection.

**Table 2:** **The titer of the neutralizing antibody against PEDV in the sera of vaccinated mice**

Antigen	Adjuvant	Dose	Number	Titre of neutralizing antibody						
				No. 1	No. 2	No. 3	No. 4	No. 5	No. 6	Geometric mean titer
PEDV/S(1–1320) + CMP + Tags	IMS1313	3	6	64	256	128	256	128	1024	203

Sera were collected from the mice vaccinated with PEDV/S(1–1320) + CMP + Tags at 42 days post-vaccination, and a serum neutralization assay was performed using Vero cells to determine the titer of the neutralizing antibody against PEDV.

## Discussion

PEDV causes major economic losses to the swine industry due to its high infectivity and mortality in neonatal piglets. Therefore, vaccination of pigs is a logical strategy for its prevention. In this study, we have designed the trimerized PEDV S protein with high immunogenicity by genetically fusing CMP trimeric motif and mass-produced using silkworm-BEVS. Induction of stable artificial trimer formation by the addition of CMP greatly improved the protein expression in silkworm larvae compared to the S ectodomain, which is difficult to self-assemble. In mice administered with the trimerized PEDV S proteins, S protein-specific IgG and neutralizing antibodies were generated in their sera. These results demonstrated that silkworm-BEVS is expected to be a very promising production system for developing subunit vaccines against coronaviruses, including PEDV.

Using our powerful silkworm-BEVS, we successfully purified about 2 mg of the antigenic PEDV S protein trimer containing both S1 and S2 domains from 10 mL of silkworm serum. This expression level is much higher than that of only the S1 domain of PEDV expressed using Spodoptera frugiperda-9 cells (3.9 mg/L), and is likely to be produced inexpensively [39]. When the full-length S protein including the transmembrane domain was expressed by BEVS in a commercial silkworm F1 hybrid, about 17 μg of protein per pupa could be expressed, but oral administration of this pupa failed to induce a significant PEDV-specific immune response in piglets [40]. On the other hand, the full-length S protein displayed on the recombinant baculovirus elicited higher neutralizing antibodies than the S1 protein, but mass production of recombinant viruses remains a technical problem [41].

In this study, based on the example of successful trimerization of gp140 of human immunodeficiency virus 1 and Fiber-knob domain of egg-drop syndrome virus [38, 42], CMP was fused as a trimerization motif to form homotrimers that mimic the native structure and also improve the expression of S protein in silkworm serum. Intermolecular disulfide bonds in the N-terminal region of CMP are thought to stabilize the oligomer and contribute to the immunogenicity of the antigen fused [31]. In other examples of the use of trimerization motifs, T4 fibritin and its deletion derivative, foldon, have been employed to trimerize the PEDV S protein for cryo-EM analyses [22, 43]. When foldon was used for trimerization, a simple analysis by non-reducing SDS-PAGE showed that a portion of the S protein remained in the trimeric state even after heating at 95 °C for 5 min, suggesting that it contributes to the stability of the S protein [44]. In the analysis using DLS, the formation of large aggregates of S protein fused with CMP was not observed even after heating at 90 °C for 5 min. These results suggest that the CMP fusion increases the expression of the S protein in the silkworm by providing high stability to the S protein and allowing proper folding. For the rational design of coronavirus spike proteins, further studies are needed to determine which trimerization motifs have the best properties in terms of solubility, stability, and immunogenicity.

In the production of recombinant proteins using silkworm-BEVS, it has been reported that the expression level of the protein varies greatly depending on the strain used. The previous researches showed the differences in the sensitivity to both *Autographa californica* nucleopolyhedrovirus (AcNPV) from 163 silkworm strains and BmNPV from the 12 strains [27, 45]. Therefore, we conducted a small-scale screening using a group of strains maintained at Kyushu University with a relatively good track record in recombinant protein production and found n22 and xa50 as suitable silkworm strains for producing PEDV spike proteins. The reasons for these differences in expression levels may be based on the diversity of immune responses and differences in viral susceptibility to BmNPV, but the differences in expression levels depending on the target protein species cannot be explained. For example, the d17 strain showed high expression of intracellularly accumulated proteins such as luciferase and DsRed, but not of PEDV S protein in the serum of this strain [27]. The n22 strain identified in this study also shows high expression of mouse interleukin-4, a secreted protein similar to S protein [25]. As mentioned above, n22 and xa50 are inbred strains and are not suitable for mass breeding. Therefore, the strains n22 and xa50 were crossed to increase the body size and disease resistance, and in addition, we expected to increase the expression of the S protein of PEDV, but the expression level of the hybrids was almost the same as that of the parent strain. However, the increase in size and resistance to disease due to hybrid vigor was remarkable, indicating that it would be helpful for mass rearing and efficient protein production.

In this study, the immunogenic analysis of the trimerized S protein showed that the protein elicited high levels of the S protein-specific IgG and the neutralizing antibodies against PEDV infection. In a previous study, three doses of a crude plant extract containing 18.76 μg of trimerized CO-26K equivalent epitopes induced a geometric mean neutralizing antibody titer of 57.6 in mouse serum, which was comparable to that of a commercial vaccine [14]. In addition, administration of the B-cell epitope of PEDV presented on hepatitis B virus-like particles at 20 μg dosage resulted in average neutralizing antibody titers between 20 and 30 [46]. Using the same neutralization method, the average titer of neutralizing antibodies in pigs inoculated with PEDV was about 128 at a viral titer of 5.62 TCID_50_/mL, and in that study, neutralizing activity was considered positive when the neutralizing antibody titer was 8 or higher [47]. Although there are some differences in these assay protocols, the geometric mean neutralizing antibody titer of 203 in this study suggests the potential of the S protein as a vaccine candidate. Because neonatal piglets with immature immune systems are highly susceptible to PEDV, a passive immunity through colostrum and milk from vaccinated sows effectively prevents them from the infection [48]. Additional studies are needed to determine whether the trimeric S protein produced in this study can induce secretory IgA antibodies necessary for passive immunity in the vaccinated sow herd.

In summary, this study describes the improved expression of the PEDV spike protein in silkworm larvae by fusing the CMP trimerization motif and using a suitable silkworm strain. The immunogenicity of the S protein was confirmed by immunization experiments in mice, and sufficient neutralizing antibodies were induced in the sera. These results indicate that the silkworm is capable of producing relatively large oligomeric proteins, and the stabilized trimeric S protein might be a promising subunit vaccine candidate against PEDV.

## Supplementary Information

**Additional file 1. The amino acid sequence of PEDV/S(1–1320) + CMP + Tags.** The sequences corresponding to PEDV spike protein (1–1320 amino acids) and the CMP were indicated as orange and blue, respectively.

**Additional file 2. ELISA analysis of serum dilutions at low doses and frequency of administration.** Female Balb/c mice (*N* = 6) were inoculated with 1 or 10 μg of purified PEDV/S(1–1320) + CMP + Tags in a micro-emulsion adjuvant (IMS1313) and boosted at 14 days after prime vaccination. At 21 days after prime vaccination, the sera of each vaccinated group were pooled and used for antigen-specific IgG ELISA. As a negative control, the group vaccinated only micro-emulsion adjuvant was used. The data were represented as mean ± standard error of the mean (in triplicate).

## Data Availability

The datasets supporting the conclusions of this article are included within the article and its additional files.

## Abbreviations

PEDV: porcine epidemic diarrhea virus
S: spike
silkworm-BEVS: silkworm-baculovirus expression vector system
M: membrane
E: envelope
N: nucleocapsid
ApN: porcine aminopeptidase N
ACE2: angiotensin-converting enzyme 2
CEACAM1: carcinoembryonic antigen-cell adhesion molecule
DDP4: dipeptidyl peptidase
COE: CO-26K equivalent epitope
BmNPV: *Bombyx mori* nucleopolyhedrovirus
POI: proteins of interest
BmO2: NIAS-Bm-Oyanagi2
CMP: Cartilage matrix protein
dpi: days post-infection
PBS: phosphate-buffered saline
CBB: Coomassie Brilliant Blue
DLS: dynamic light scattering
OD: optical density
CPE: Cytopathic effect
TM: transmembrane
AcNPV: *Autographa californica* Nucleopolyhedrovirus
dpv: days post-vaccination
