# Supplementary material for: Stable trimer formation of spike protein from porcine epidemic diarrhea virus improves the efficiency of secretory production in silkworms and induces neutralizing antibodies in mice
Source: Vet Res. 2021 Jul 7;52:102. doi: 10.1186/s13567-021-00971-5 (PMC8261802; doi:10.1186/s13567-021-00971-5)
Supplement: Supplementary file 1 — Additional file 1. The amino acid sequence of PEDV/S(1–1320) + CMP + Tags. The sequences corresponding to PEDV spike protein (1–1320 amino acids) and the CMP were indicated as orange and blue, respectively. [file 13567_2021_971_MOESM1_ESM.pptx]

## Slide 1
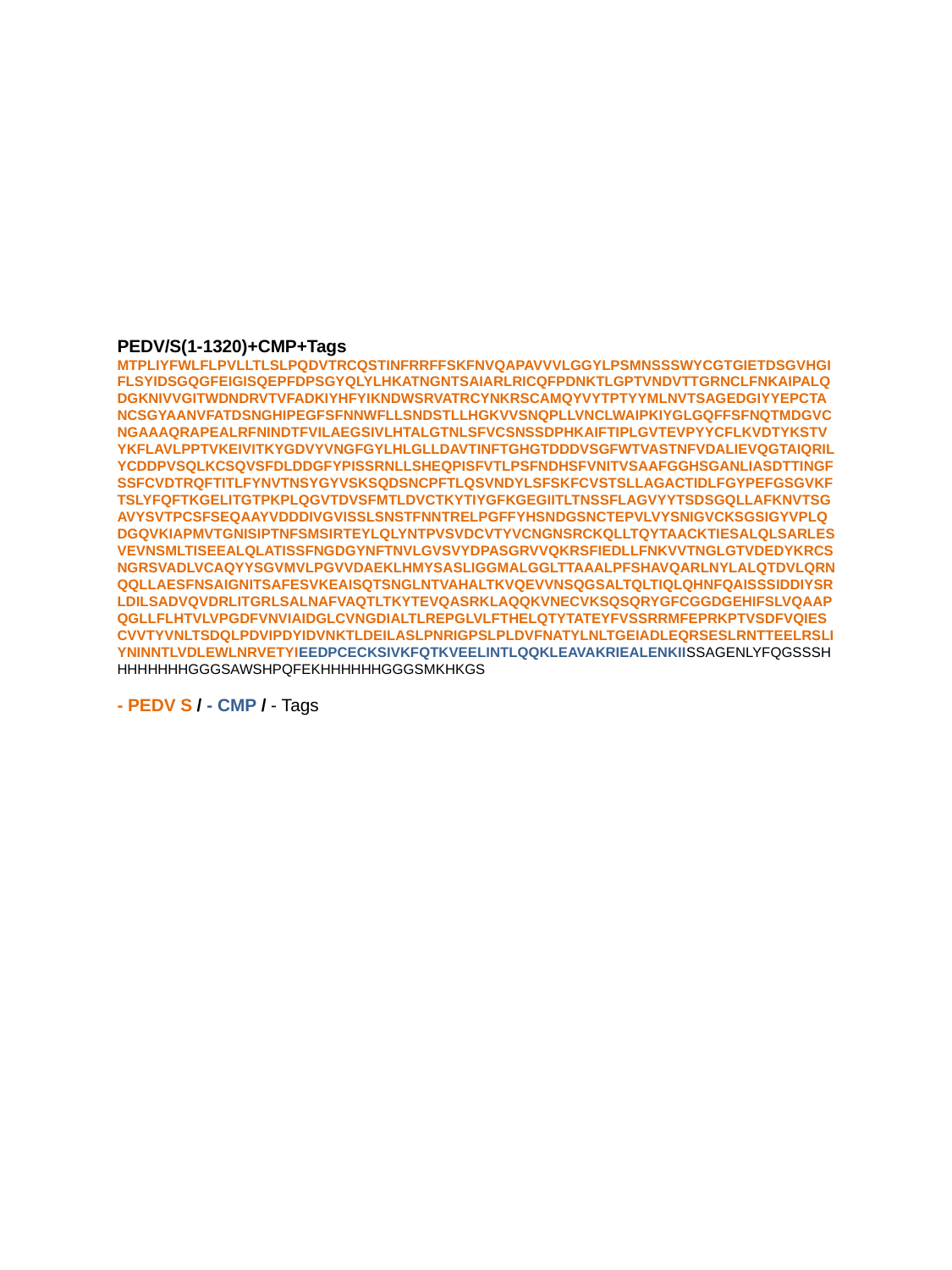

PEDV/S(1-1320)+CMP+Tags
MTPLIYFWLFLPVLLTLSLPQDVTRCQSTINFRRFFSKFNVQAPAVVVLGGYLPSMNSSSWYCGTGIETDSGVHGIFLSYIDSGQGFEIGISQEPFDPSGYQLYLHKATNGNTSAIARLRICQFPDNKTLGPTVNDVTTGRNCLFNKAIPALQDGKNIVVGITWDNDRVTVFADKIYHFYIKNDWSRVATRCYNKRSCAMQYVYTPTYYMLNVTSAGEDGIYYEPCTANCSGYAANVFATDSNGHIPEGFSFNNWFLLSNDSTLLHGKVVSNQPLLVNCLWAIPKIYGLGQFFSFNQTMDGVCNGAAAQRAPEALRFNINDTFVILAEGSIVLHTALGTNLSFVCSNSSDPHKAIFTIPLGVTEVPYYCFLKVDTYKSTVYKFLAVLPPTVKEIVITKYGDVYVNGFGYLHLGLLDAVTINFTGHGTDDDVSGFWTVASTNFVDALIEVQGTAIQRILYCDDPVSQLKCSQVSFDLDDGFYPISSRNLLSHEQPISFVTLPSFNDHSFVNITVSAAFGGHSGANLIASDTTINGFSSFCVDTRQFTITLFYNVTNSYGYVSKSQDSNCPFTLQSVNDYLSFSKFCVSTSLLAGACTIDLFGYPEFGSGVKFTSLYFQFTKGELITGTPKPLQGVTDVSFMTLDVCTKYTIYGFKGEGIITLTNSSFLAGVYYTSDSGQLLAFKNVTSGAVYSVTPCSFSEQAAYVDDDIVGVISSLSNSTFNNTRELPGFFYHSNDGSNCTEPVLVYSNIGVCKSGSIGYVPLQDGQVKIAPMVTGNISIPTNFSMSIRTEYLQLYNTPVSVDCVTYVCNGNSRCKQLLTQYTAACKTIESALQLSARLESVEVNSMLTISEEALQLATISSFNGDGYNFTNVLGVSVYDPASGRVVQKRSFIEDLLFNKVVTNGLGTVDEDYKRCSNGRSVADLVCAQYYSGVMVLPGVVDAEKLHMYSASLIGGMALGGLTTAAALPFSHAVQARLNYLALQTDVLQRNQQLLAESFNSAIGNITSAFESVKEAISQTSNGLNTVAHALTKVQEVVNSQGSALTQLTIQLQHNFQAISSSIDDIYSRLDILSADVQVDRLITGRLSALNAFVAQTLTKYTEVQASRKLAQQKVNECVKSQSQRYGFCGGDGEHIFSLVQAAPQGLLFLHTVLVPGDFVNVIAIDGLCVNGDIALTLREPGLVLFTHELQTYTATEYFVSSRRMFEPRKPTVSDFVQIESCVVTYVNLTSDQLPDVIPDYIDVNKTLDEILASLPNRIGPSLPLDVFNATYLNLTGEIADLEQRSESLRNTTEELRSLIYNINNTLVDLEWLNRVETYIEEDPCECKSIVKFQTKVEELINTLQQKLEAVAKRIEALENKIISSAGENLYFQGSSSHHHHHHHHGGGSAWSHPQFEKHHHHHHGGGSMKHKGS
- PEDV S / - CMP / - Tags
