# Supplementary figures and images for: Stable trimer formation of spike protein from porcine epidemic diarrhea virus improves the efficiency of secretory production in silkworms and induces neutralizing antibodies in mice
Source: Vet Res. 2021 Jul 7;52:102. doi: 10.1186/s13567-021-00971-5 (PMC8261802; doi:10.1186/s13567-021-00971-5)

## Slide 1
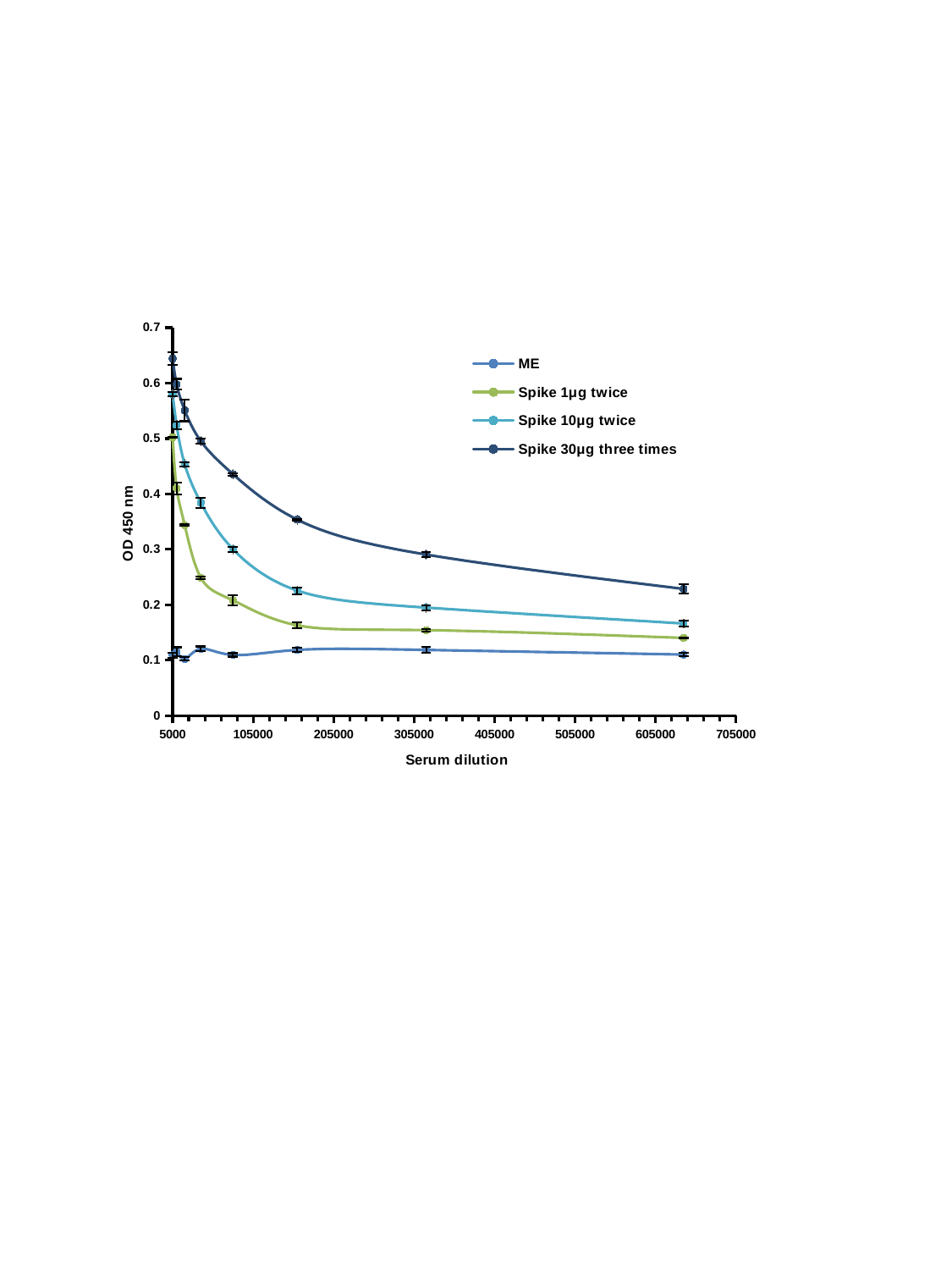

### Chart
| Category | ME | Spike 1μg twice | Spike 10μg twice | Spike 30μg three times |
|---|---|---|---|---|

Supplement: Supplementary file 2 — Additional file 2. ELISA analysis of serum dilutions at low doses and frequency of administration. Female Balb/c mice (N = 6) were inoculated with 1 or 10 μg of purified PEDV/S(1–1320) + CMP + Tags in a micro-emulsion adjuvant (IMS1313) and boosted at 14 days after prime vaccination. At 21 days after prime vaccination, the sera of each vaccinated group were pooled and used for antigen-specific IgG ELISA. As a negative control, the group vaccinated only micro-emulsion adjuvant was used. The data were represented as mean ± standard error of the mean (in triplicate). [file 13567_2021_971_MOESM2_ESM.pptx]
